# Genome-Wide Analysis, Evolutionary History and Response of *ALMT* Family to Phosphate Starvation in *Brassica napus*

**DOI:** 10.3390/ijms22094625

**Published:** 2021-04-28

**Authors:** Ismail Din, Ihteram Ullah, Wei Wang, Hao Zhang, Lei Shi

**Affiliations:** 1 National Key Laboratory of Crop Genetic Improvement, Huazhong Agricultural University, Wuhan 430070, China; IsmailDin@webmail.hzau.edu.cn (I.D.); weiwfftd2017@webmail.hzau.edu.cn (W.W.); 2 Key Laboratory of Arable Land Conservation (Middle and Lower Reaches of Yangtze River), Microelement Research Centre, Ministry of Agriculture and Rural Affairs, Huazhong Agricultural University, Wuhan 430070, China; hao.zhang@webmail.hzau.edu.cn; 3 Department of Plant Breeding and Genetics, Gomal University, Dera Ismail Khan 29220, Pakistan; ihterampbg@gu.edu.pk

**Keywords:** *Brassica napus*, ALMT gene family, phylogenetic analysis, phosphorus-starvation, gene expression

## Abstract

Low phosphorus (P) availability is one of the major constraints to plant growth, particularly in acidic soils. A possible mechanism for enhancing the use of sparsely soluble P forms is the secretion of malate in plants by the aluminum-activated malate transporter (ALMT) gene family. Despite its significance in plant biology, the identification of the *ALMT* gene family in oilseed rape (*Brassica napus*; *B. napus*), an allotetraploid crop, is unveiled. Herein, we performed genome-wide identification and characterization of *ALMTs* in *B. napus*, determined their gene expression in different tissues and monitored transcriptional regulation of *BnaALMTs* in the roots and leaves at both a sufficient and a deficient P supply. Thirty-nine *BnaALMT* genes were identified and were clustered into five branches in the phylogenetic tree based on protein sequences. Collinearity analysis revealed that most of the *BnaALMT* genes shared syntenic relationships among *BnaALMT* members in *B. napus*, which suggested that whole-genome duplication (polyploidy) played a major driving force for *BnaALMTs* evolution in addition to segmental duplication. RNA-seq analyses showed that most *BnaALMT* genes were preferentially expressed in root and leaf tissues. Among them, the expression of *BnaC08g13520D*, *BnaC08g15170D*, *BnaC08g15180D*, *BnaC08g13490D*, *BnaC08g13500D*, *BnaA08g26960D*, *BnaC05g14120D*, *BnaA06g12560D*, *BnaC05g20630D*, *BnaA07g02630D*, *BnaA04g15700D* were significantly up-regulated in *B. napus* roots and leaf at a P deficient supply. The current study analyzes the evolution and the expression of the ALMT family in *B. napus*, which will help in further research on their role in the enhancement of soil P availability by secretion of organic acids.

## 1. Introduction

Phosphorus (P) is an essential nutrient for plant growth and development and is involved in a number of metabolic processes [1]. Although total P in the soil is plentiful yet, soluble P (Pi) is often low [2], mainly because a large amount of P in soil is fixed by metal oxides (acidic soil) or carbonate compounds (alkaline soil), or exists in the form of organic P [3,4]. P deficiency has been one of the main constraints in agricultural production, restricting crop yields of arable land worldwide from 30% to 40% [5]. The plant root can consume only Pi, which normally is present on a micro-molar basis in soils [6]. Plants have established a variety of adaptive strategies in response to P deficiency. These include enhancement of soil Pi availability through rhizosphere acidification by organic acid secretion (OA) and secretion of hydrolytic enzymes (Apase, RNase and Phytase); increasing Pi uptake capability by changing the root system architecture, raising the abundance of high-affinity Pi transporters and establishing associations with arbuscular mycorrhizal (AM); improving physiological P consumption performance through reducing organic P pools by alternate metabolic pathway lipid remodeling and increasing P redistribution from senescing tissues to developing tissues, sustaining yield by maximizing light capture and seed yield biomass allocation [7,8,9].

An aluminum-activated malate protein transporter (ALMT) plays a crucial role in the adaptation under acidic soil conditions [10]. In several organisms, numerous *ALMT* homologous have been cloned and extensively characterized [11]. For example, *TaALMT1* regulates the malate-activated aluminum efflux from the bread wheat roots [12]. The key problem of acid soil is the increased mobility of aluminum ions and their ability to form highly stable phosphorous complexes. As a result, the plant faces not only the toxicity of Al^3+^ ions but also the low bioavailability of Pi [13,14]. Both issues are solved by releasing organic acids, which could chelate Al^3+^ ions and release Pi [13,14]. *ALMT* proteins are regulated at transcriptional and functional levels [13,14]. The existing lack of a crystal structure for modelling the structure of ALMT transporters has impeded the understanding of the structure-function which are the fundamental aspect of ALMT-mediated transportation processes [15,16]. A sequence-based analysis showed that as a family, ALMT has a high level of secondary structural similarity with an *n*-terminus region containing 6 transmembrane domains, and a long, strongly hydrophilic C-terminus of up to half the length of the whole protein [15,17]. Furuichi et al. (2010) [18] have identified three key residues (Glu274, Asp275 and Glu284) that eliminate Al-dependent transport changes after neutralization, without affecting the basal transport activity; they have concluded that these residues are likely to be in the extracellular C-terminal region as a major determinant of Al^3+^-activation response [19]. The high level of conservation of these residues demonstrates their value for structural integrity [15,17].

*Brassica napu* (*B. napus*, AC genome) is a domesticated allotetraploid, arising from the natural hybridization of the diploid species *Brassica rapa (B. rapa*, A genome) and *Brassica oleracea* (*B. oleracea*, C) over 7500 years ago. It is the third largest source of vegetable oil globally. The lack of plant-available Pi in soil could impede the development and growth of *B. napus* and reduce its production and quality in return [20]. Citric and malate could be secreted by phosphate-starved oilseed rape to improve soil P mobilization capacity [21]. *B. napus* had rapid rates of carboxylic acid exudation (Citric acid), which together with its greater root length and root surface area, could allow the plant to acquire poorly available P forms and explore a larger soil volume that facilitate P uptake [22], there have been limited studies on the adjustment to P starvation for the *ALMT* gene family in *B. napus*. In this study, we identified the members of the *BnaALMT* family, their characterization, intrinsic diversity, heterogeneity of expression and functional divergence under contrasting P supplies. 

## 2. Results

### 2.1. Identification of the ALMT Family Genes in B. napus

A total of 14 *ALMT* family members were identified in Arabidopsis. The *ALMT* family genes in *B. napus* were retrieved from *Brassica* databases (BRAD, http:/brassicadb.org/brad; accessed on 11 April 2020) using *AtALMTs* as reference. A high similarity among the orthologues of *AtALMTs* and *BnaALMTs* was observed in most of the cases. Some of the *B. napus* protein sequences were up to 75% similar to their corresponding orthologues in *Arabidopsis*, while others similarity merely ranged from 40% to 50%. According to the selected threshold value of alignment similarity 75%, we found a total of thirty-nine (39) *ALMTs* in the *B. napus* genome. All 39 homologous of the 14 members of the *ALMT* family, were further confirmed by the published online “Zhongshuang-11” *B. napus* transcriptomic databases (CRA001775) from the National Genomics Data Centre [23].

### 2.2. Evolutionary and Phylogenetic Relationship between Arabidopsis and B. napus ALMTs

**Figure 1:**
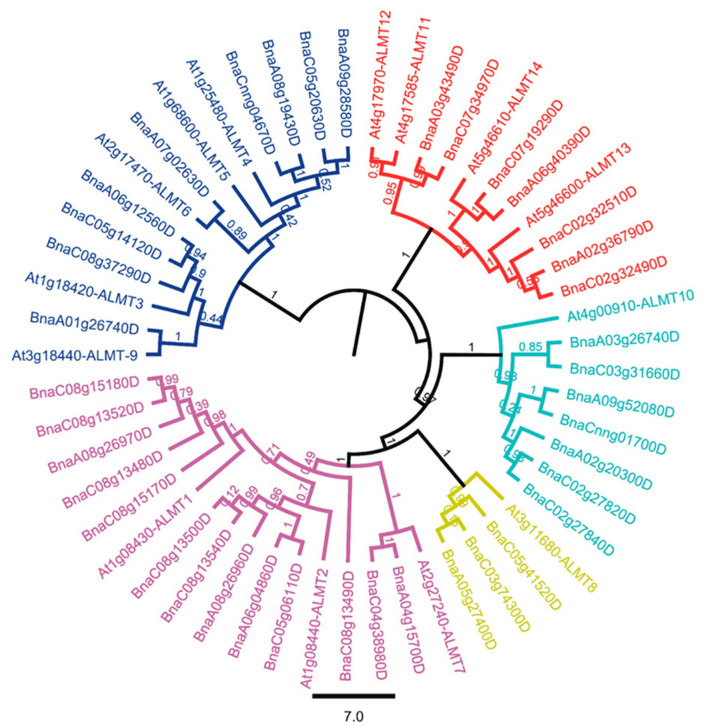
Phylogenetic tree of aluminum malate transporter (*ALMT*) genes family in *Brassica napus* and *Arabidopsis thaliana*. The phylogenetic tree was constructed by Genius software. The tree consists of five clades, different colors of the clades in the tree represent homologous genes of *Brassica napus* and *Arabidopsis thaliana*. The analysis involved 53 nucleotide sequences including 39 from *Brassica napus* and fourteen from *Arabidopsis*.

To establish the phylogenetic relationships among the *BnaALMT* family genes and *AtALMTs*, we aligned the protein sequences of all 39 *BnaALMT* genes with 14 *AtALMT* using MAFFT, and the phylogenetic tree was constructed based on multi-alignment using FastTree plugin in Geneious 11. Based on its homology with fourteen members in *Arabidopsis*, the alignment and eventual construction of the *ALMT* genes in *B. napus* was established. Based on the genes’ relationship, the phylogenetic tree was divided into five distinct clades (Figure 1). Clade-1 was split into two subclades and 14 genes; Sub-clade-1 comprised six genes (*At3g18440*, *BnaA01g26740D*, *At1g18420*, *BnaC08g37290D*, *BnaC05g14120D* and *BnaA06g12560D*) while subclade-2 comprised eight genes (*At2g17470*, *BnaA07g02630D*, *At1g68600*, *At1g25480*, *BnaA09g28580D*, *BnaC05g20630D*, *BnaA08g19430D* and *BnaCnng04670D*). *ALMT5* could not align (*At1g68600*) with any *BnaALMT* family member homologs. Clade-2 consisted of 11 genes and was divided into three subclasses comprising genes (*At4g17585*, *At4g17970*, *BnaA03g43490D*, *BnaC07g34970D*, *At5g46610*, *BnaC07g19290D*, *BnaA06g40390D*, *At5g46600*, *BnaC02g32510D*, *BnaA02g36790D* and *BnaC02g32490D*). It is worthy to note that *AtALMT11* and *AtALMT12* form parallel lineage to each other which might have arisen through gene duplication event, in *Arabidopsis*. Clade-3 consisted of eight genes (*At4g00910*, *BnaA03g26740D*, *BnaC03g31660D*, *BnaA09g52080D*, *BnaCnng01700D*, *BnaA02g20300D*, *BnaC02g27820D* and *BnaC02g27840D*), and Clade-4 included four genes (*At3g11680*, *BnaC05g41520D*, *BnaC03g74300D* and *BnaA05g27400D*). Further, Clade-5 was the largest clade of all which contained 16 genes and split into three subclades (*At2g27240D*, *BnaA04g15700D*, *BnaC04g38980D*, *BnaC08g13490D*, *At1g08440D*, *BnaC05g06110D*, *BnaA06g04860D*, *BnaA08g26960D*, *BnaC08g13540D*, *BnaC0813500D*, *At1g08430D*, *BnaC08g15170D*, *BnaC08g13480D*, *BnaA08g26970D*, *BnaC08g13520D* and *BnaC08g15180D*). *BnaC08g13490D* was stood alone and not grouped with any members of the *AtALMT* family genes.

### 2.3. Gene Structure and Conserved Motif Analysis of the BnaALMT Genes Family

**Figure 2:**
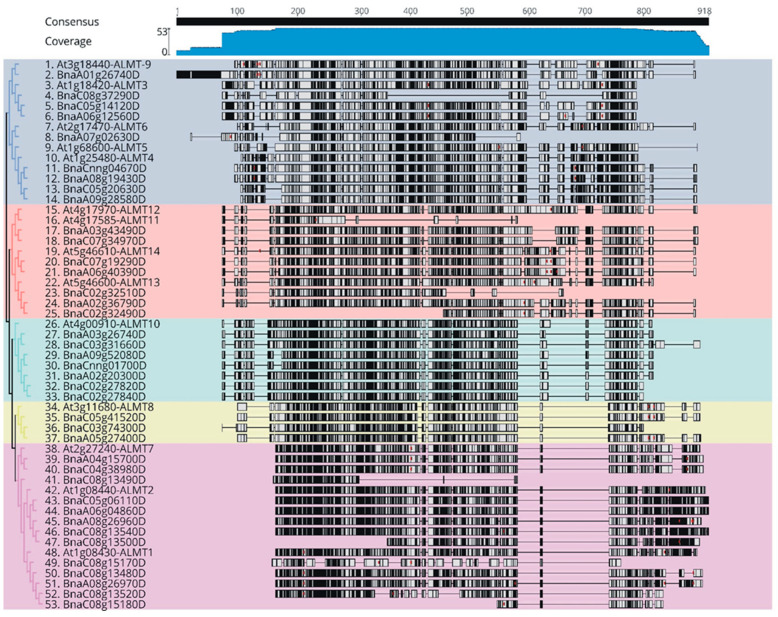
The variation distribution of *ALMT* genes in the phylogenetic relationship was constructed by Genius 11. Sequence similarities among the *ALMT* genes family of the *Brassica napus* and *Arabidopsis thaliana* were aligned using MUSCLE (a new computer program for creating multiple alignments of protein sequences) alignment values. *Brassica napus* and *Arabidopsis thaliana ALMTs* were divided into five groups based on FastTree. Black boxes indicate sequence similarities and white boxes represent dissimilarities among the *ALMT* genes family. The length of the scale represents base pairs (bp) length which is placed at the top.

To gain an understanding of the phylogenetic relationship of critical sequence polymorphisms among the *BnaALMT* family genes with *AtALMTs* in their flanking areas, we analyzed the gene structure by comparing the protein sequences of *BnaALMTs* based on their gene information from the GENOSCOPE database. For the analysis of protein sequence variation, we had blasted the protein sequences of 39 *BnaALMT* gene families with 14 *AtALMT* members. In *B. napus* homologs of the *ALMT* families which were in adjacent branches of the phylogenetic tree, the same gene structure was exhibited (Figure 2), which could then divide the phylogenic variation of these *ALMT* genes into 5 independent classes. Among them, *Class 1* had ten *BnaALMT* genes, *Class 2* and *Class 3* both had seven *BnaALMT* genes, *Class 4* had three *BnaALMT* genes and *Class 5* had 13 *BnaALMT* genes. Moreover, within each class *BnaALMT* genes and *AtALMTs* were highly variable and were arranged in different sequence lengths and numbers. For example, the length of the conserved sequence, the dissimilarities and the deletion of the *BnaALMT* genes ranged greatly among the various members of the *BnaALMT* gene family, varying between 115 and 666 bp, with numbers ranging from 1 to 918 bp lengths at top of the scale (Figure 2). The *BnaALMT* genes in *Class1* were lengthier and had much more dissimilar regions in their homologues, whereas the *BnaALMT* genes in *Class5* have more conserved gene regions than other classes. However, the deletion regions among the *BnaALMT* genes of *Class 3*, *Class 4* and *Class 5* were the same, varying between 600 to 800 bp lengths at top of the scale (Figure 2). In comparison, some of the genes had lost their protein sequences in various groups.

The orthological gene pairs between *A. thaliana* and *B. napus* were traced, with protein structural models for *BnaALMTs* aligned to *AtALMTs* generated with the MAFFT sequences used by the *Arabidopsis thaliana* family and *B. napus ALMT* family genes (Figure S1). *BnaALMTs* were much different in structures than *AtALMTs*. The findings of several sequences of alignment showed that the protein structural diversity of *B. napus* and *A. thaliana ALMT* genes (Figure S1) was obvious from *Clade1*, *Clade2*, *Clade3*, *Clada4* and *Clade5*. Beta-strand paired with *AtALMTs* had been formed in the *BnaALMT* family sequence alignments, such as *AtALMT6*, *AtALTM7*, *AtALMT11*, *AtALMT12*, *AtALMT13* and *AtALMT14* homologous. The analysis of the gene structure of the *ALMT* families in Arabidopsis and *B. napus* indicated that the gene structure differed across various subgroups but was conserved in the same subgroup.

**Figure 3:**
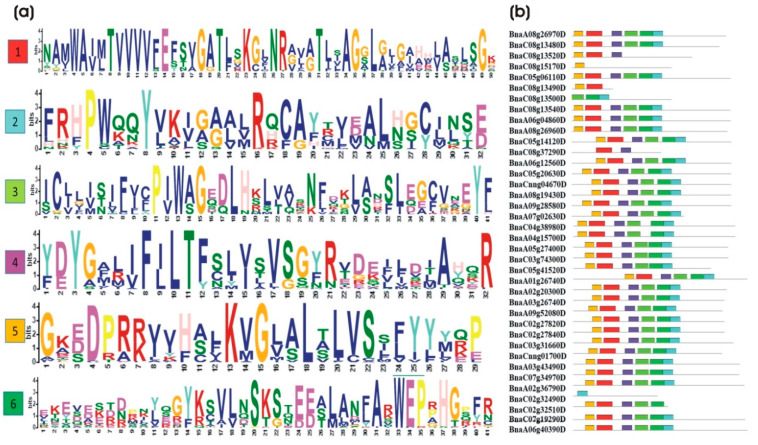
Distribution of conserved motifs in *BnaALMTs*. Conserved motifs of *BnaALMTs* were analyzed by using MEME-suite Web serve using the protein sequences of thirty-nine *BnaALMTs*. Six conserved motifs were identified, and different motifs were distinguished by different colors. (**a**) The conserved motifs among *BnaALMT* proteins. The least motif of six contains WEP (Trp. Glu. Pro.) conserved sequences, which are conserved in all *BnaALMT* genes except one, and one gene is completely lost during sequence analysis. (**b**). The distribution of motifs along with the protein sequences. Thirty-one *BnaALMT* genes share all the six motifs, while few genes have lost the other domains, respectively. Only two genes have retained the single motif but have lost the other domains completely during evolution.

To identify a common motif among these proteins, all 39 genes of *BnaALMT* were subjected to the online MEME (http:/meme-suite.org/; accessed on 17 April 2020) tool. The analysis was conducted with the “motifs search” while the rest of the setting was maintained as default. A total of 6 motifs in the majority of *B. napus ALMT’s* family protein were identified in MEME suits. We found several highly conserved sequences including TVVVVFE, AG(X)L, PW(X)(X)(X)Y, R(X)CA, P(X)W(X)G, K(X)G(X)(X)L(X)LVS, F(X)LTF, and WEP in protein sequences of *BnaALMTs*. However, here we did not find any of their reported functions in literature except for the WEP sequence and the sequences highlighted from the motifs 3 and 6 residues which were highly similar to the earlier study in wheat. All of these sequences had significant functionality as they were evolutionary similar to their orthologues in different species. Most of the *BnaALMTs* contained 6 motifs except for *BnaC08g15170D*, *BnaC08g133500D* and *BnaC08g37290D* (Figure 3b). The *BnaALMT* proteins responsible for DNA binding were conserved in a motif-1. Motif-3 and Motif-5 were rich in G (glycine). Rich in valine (V) and glycine (G) were usually associated with dimerization of proteins. In *BnaALMTs*, the presence of G-rich motifs showed that these proteins could interact with several other proteins and thus make *BnaALMTs* better able to integrate with various biological processes in *B. napus*. Motif-2, Motif-4 and Motif-6 were highly variable among *BnaALMT* proteins which could be responsible for various biological functions in these *BnaALMTs*. The highly variable motif showed a wide range of functional diversity for *BnaALMTs*. Contractions between motif 2 and 6 in the *ALMT* genes that were likely due to segment deletion in these genes, were also evident in protein structures. Motif-6 included a WEP conserved sequence that was part of the standard fingerprint pattern (Trp-Glu-Pro) found in all *ALMTs* to evaluate the *ALMT* functions.

A broad range of functional diversities for *BnaALMT* was indicated by the presence of highly variable motifs. Some of the patterns of the *BnaALMT* genes were well spread in an exact position of sequences, whereas the other *BnaALMT* genes were placed in a pattern of an abnormal protein sequence (Figure 3b). The same lineage might have come from *BnaC08g13490D* and *BnaC08g13500D*, while these two genes had been split due to the deletion of the segment of genes. Apparent contractions were also found in the protein structures between motif 1 to 6 in *BnaC02g32510D*, *BnaC08g13520D* and *BnaC08g37290D* which was possibly the product of segment deletions in these genes which had lost one, three and four motifs, respectively (Figure 3b). The gene *BnaC08g15180D* was completely lost all the motifs (1 to 6). The genes of *BnaC08g15170D* and *BnaC02g32490D* were possible pseudogenes, with the loss or the gain of part of the A genome sections being able to result in protein expansion and contraction. In each subgroup, most closely related genes had a similar motif composition, although the motif composition varied significantly among the various subgroups. The similar motif arrangements between *ALMT* protein sub-groups indicated that in one specific subgroup the protein architecture was preserved. Overall, together with the results of phylogenetic analysis, the similar gene structure and conserved motif composition of *ALMT* members of the same group strongly supported the confidence of group classification.

### 2.4. Chromosomal Distribution and Duplication of the BnaALMT Genes

**Figure 4:**
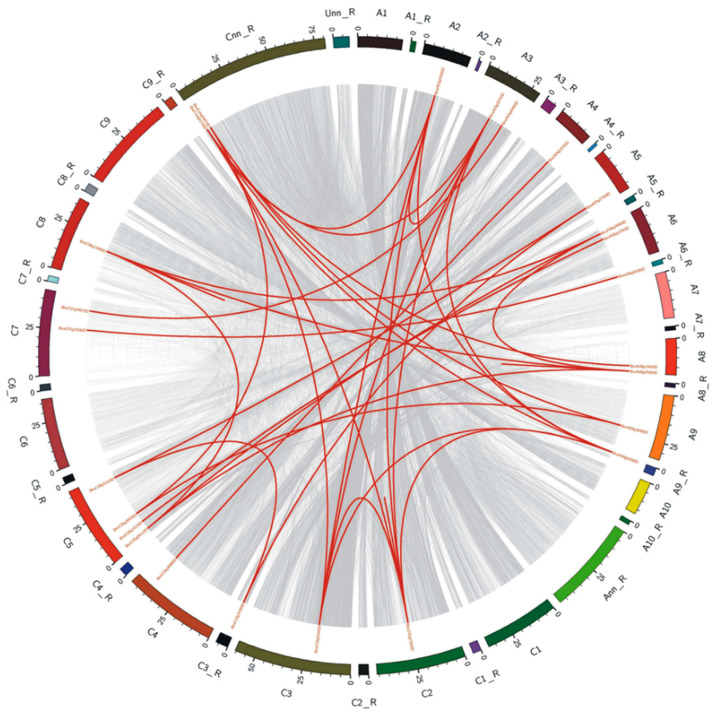
Schematic representations for the chromosomal distribution and interchromosomal relationships of *Brassica napus ALMT* genes. Grey lines indicate all syntenic blocks in the *Brassica napus* genome, and the blue lines indicate syntenic *ALMT* gene pairs. The chromosome numbers are indicated at the top of each chromosome. R, random.

**Figure 5:**
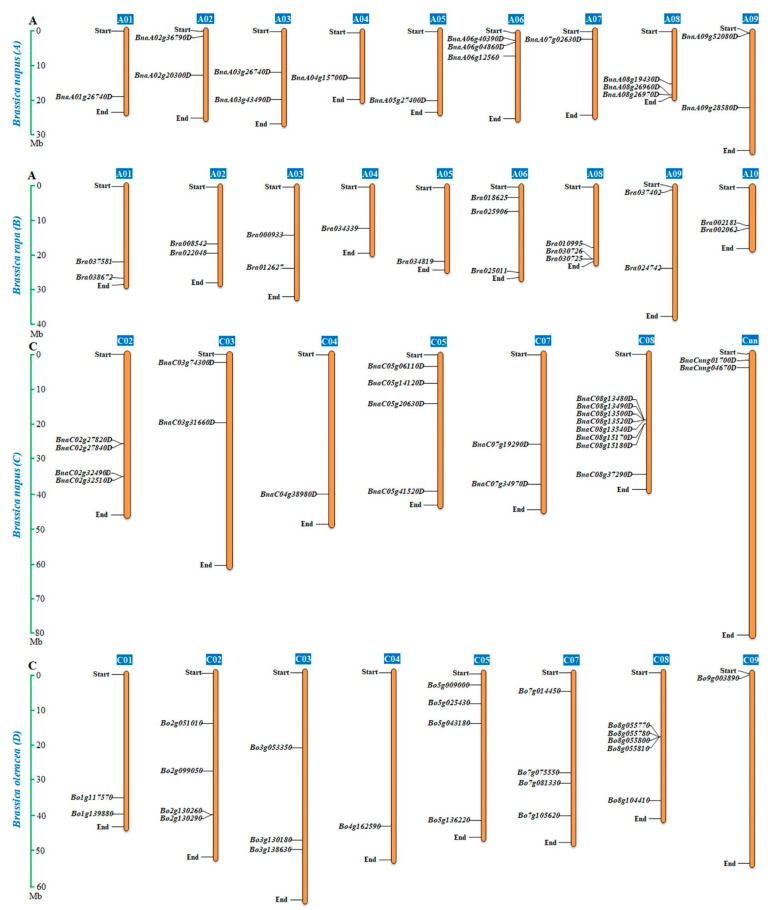
Genomic distribution of *ALMT* genes in *Brassica napus*, *Brassica rapa* and *B. oleracea* chromosomes. The *Brassica* species (*Brassica napus*, *Brassica rapa* and *B. oleracea*) *ALMT’s* were plotted based on the location of genes, length of chromosomes, and position of centromeres. Each chromosome indicated the gene density by the frequency per 1 Mb.

To analyze the evolution of *ALMTs* using a syntenic genetic study, we traced the orthologous gene pairs among *Brassica* species. A total of 25 pairs of genes in orthology were found *in B. napus* (Figure 4). In contrast with *A. thaliana*, *Brassica* species have suffered an additional whole-genome triplication (WGT) [24]. The *ALMT* genes may have triplicate orthologous copies in *B. rapa* and *B. oleracea*, as WGT from the *Brassica* ancestor in the *A. thaliana* genome. Gene duplication analysis with the syntenic, phylogenetic and chromosome-site study had shown that 39 *BnaALMT* genes were scattered unevenly across 16 chromosomes, except A01, A10, and C07, with 16 in A sub-genome and 23 in the C sub-genome (Figure 5). Two members of *BnaCnng04670D* and *BnaCnng01700D* were found in the C sub-genome, but they could not be mapped to a particular chromosome. There were comparatively some chromosomes (e.g., ChrC08) that had relatively many genes, or relatively few genes (e.g., ChrA07). Chromosome C08 had the most numerous *BnaALMT* genes and eight *BnaALMT* genes have emerged in the gene cluster. The chromosome length and number of *BnaALMT* genes had no positive association. Furthermore, both *BrALMTs* and *BoALMTs* were mapped on their chromosomes except for ChrA07 in *B. rapa*, and ChrC06 and ChrC10 in *B. oleraceae* (Figure 5).

Gene family expands mainly via three pathways; tandem duplication, segmental duplication, and whole-genome duplication [25]. A tandem duplication case is defined as a chromosomal region within 200 kb containing two or more homologous genes [26]. To further uncover the evolution processes of the *BnaALMT* family, we analyzed the gene replication patterns using their CDS sequences (Figure 3a). Here, twenty-five *BnaALMT* genes (64.1%) were divided into seven chromosome tandem replication regions on chromosomes A03, A06, A08, C05 and C07 (Figure 4). Each tandem cluster had 2–3 duplicated genes. Besides the tandem-duplication events, 129 segment-specific duplication events were identified with 39 genes in *ALMT* using BLASTP and MCScanX methods (Figure 4), suggesting that the evolution of the *BnaALMT* gene family has been powered by segment-specific duplication events. Because almost all of the *BrALMT’s* and *BoALMT’s* homologous products retained ties with *BnaALMTs*, in addition to segmental duplication, complete genome duplication (polyploidy) was also a major driving force of *BnaALMT*.

### 2.5. Expression Profile of BnaALMT Genes in Different Tissues

**Figure 6:**
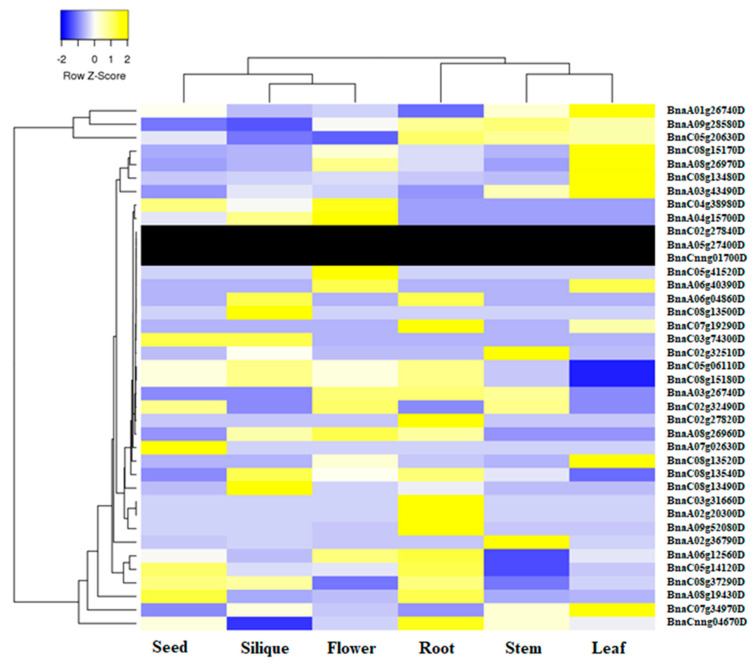
Transcript abundance of *BnaALMT* genes in various tissues of *Brassica napus*. The heatmap was constructed by taking Log2 values of the transcript per million fragments mapped (FPKM) generated from RNA-seq data. The color scale (blue-yellow) of the heatmap indicates expression values, respectively. While the black rows show that the expression of the genes was not detected in the RNAseq data. The cluster tree of the *BnaALMT* genes based on the expression level is shown on the left and top.

We used online transcription information for *B. napus* cultivar “Zhonghuang11” to define the tissue-specific expression profile of different genes in the *BnaALMT* gene family under nutrients sufficient conditions (Figure 6). In total, 18 *BnaALMT* genes homologous to *AtALMT1*, *AtALMT2*, *AtALMT3*, *AtALMT4*, *AtALMT10*, *AtALMT13* and *AtALMT14* had exceedingly high expression in root, while 11 *BnaALMT* genes homologous to *AtALMT1*, *AtALMT4*, *AtALMT9*, *ALMT11*, *AtALMT12*, and *AtALMT14* had highly expression in leaves. In addition, *BnaALMT* members showed a diverse expression pattern in other tissues including seed, silique, flower and stem. Among the tissues evaluated for expression analysis, developing seeds shared the least *BnaALMT* members. Interestingly, not a single *BnaALMT* family member was found to have high transcript abundance in all the examined tissues indicating they had highly specific roles in different tissues.

### 2.6. The Expression Pattern of BnaALMTs in Response to Phosphorus Stress

**Figure 7:**
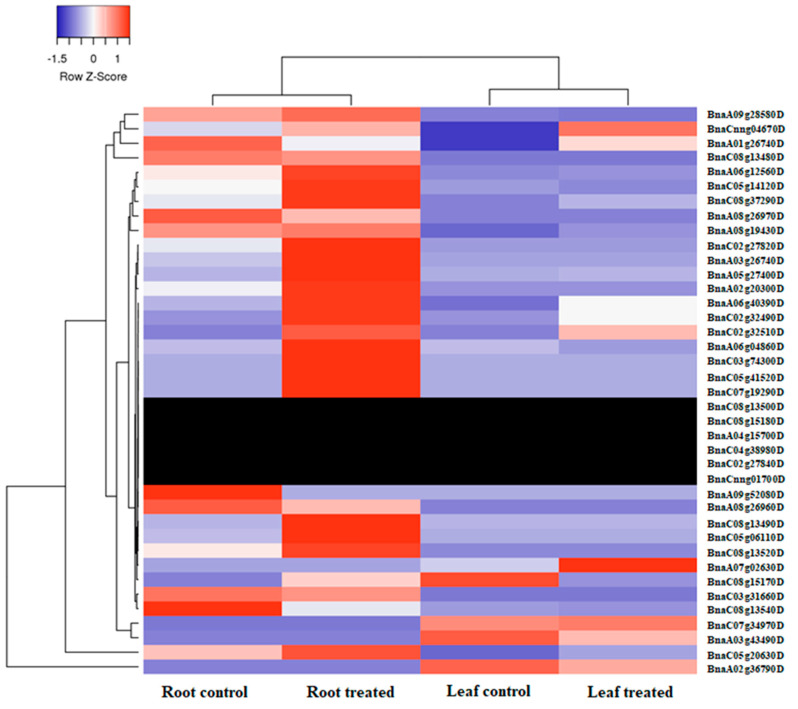
Expression profiles of the *BnaALMT* family genes in the leaves and roots of *Brassica napus* at a sufficient P supply (control) and a deficient P supply (treated) in Hoagland’s solution. The color bar represents log2 expression levels (FPKM, per kilobase of exon per million fragments mapped) of each gene. Data represent the average of three biological replicates. The color scale (blue-red) of the heatmap indicates expression values, and the dark black line represents that *BnaALMT* genes were not detected by their transcript abundance, respectively. The cluster tree of the *BnaALMT* genes based on the expression level is shown on the left and top.

**Figure 8:**
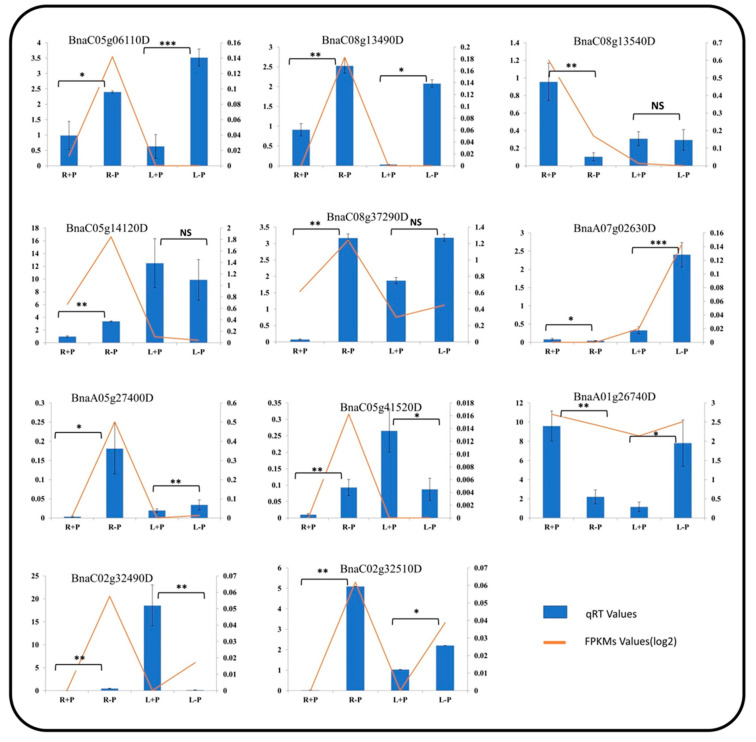
Expression patterns of the up-regulated *BnaALMTs* at a P sufficient supply and a P deficiency supply in Hoagland’s solution. Three biological replicates were employed in this study. The relative expression of *BnaALMTs* in the *Brassica napus* root and leaf under contrasting P supplies was quantified by q-PCR; The bars represent q-PCR data while the line represents RNA-seq data. Student *T*-test was applied to treated and non-treated tissues (leaves and roots) to find out the statistical differences caused by phosphorous application in the tissues. ns= non-significant, * = significant at 0.05, ** = significant at 0.01 and *** = significant at 0.001. Three biological replicates. All primer sequences are listed in supplementary Table S1.

The Leaf (L) and root (R) of the *B. napus* cultivar “Zhongshuang11” under P sufficient and deficient conditions were used to examine the transcriptional profile of *BnaALMT* genes in response to P deficiency. The heat map was based on *BnaALMT* family genes expression values (FPKMs) derived from the RNA sequence data (Figure 7). A total of thirty-nine (39) *BnaALMTs* family genes showed altered gene expression responding to P deficiency. Most *BnaALMT* genes showed up-regulated in the roots as compared to the leaves. Nearly half of the *BnaALMTs* were up-regulated after P deprivation in the roots. In the treated roots, only three of the *BnaALMT* genes had low transcriptional abundance than control samples (sufficient P supply), including *BnaA01g26740D*, *BnaA08g26970D*, *BnaC08g13540D*; and in the treated leaves, most of the *BnaALMT* genes were down-regulated, except *BnaC07g34970D*, *BnaA07g02630D*, *BnaCnng04670D* as compared to the control. Additionally, four *BnaALMT* genes (*BnaC08g13480D*, *BnaA08g19430D*, *BnaA09g28580D* and *BnaC03g31660D*) that displayed quite a similar expression were all up-regulated in roots of the cultivar “Zhongshuang11” at both sufficient and deficient P supplies. Twenty-two *BnaALMT* genes were selected for qRT-PCR assay, and the results verified that the expression pattern of them was in compliance with the RNA sequence data in Figure 8. These results indicated that the *BnaALMT* family is possibly involved in P starvation responses.

## 3. Discussion

### 3.1. Phylogenetic Tree of BnaALMTs

ALMT plays a crucial role in the adaptation under acidic soil conditions [10]. Systematic phylogenic analysis shows that the *ALMT* family present in higher plants can already be divided into five clades [17,27]. To our knowledge, there is not any report in the literature describing *BnaALMT* gene family lineage and their gene functions in detail. Genome resequencing offers an efficient means of defining a significant number of variations in *AtALMTs* and *BnaALMT* genes. A total of 39 *BnaALMT* genes in the *B. napus* genome were identified in this study (Figure 1). Of them, 75% had high similarity homologous pairs (Figure 1). These *BnaALMT* genes were classifiable into five clusters according to the phylogenetic tree. Cluster 5 was the largest cluster with 13 members, followed by Cluster 1 of nine members, Cluster 2 and Cluster 3 of seven, while Cluster 4 was the smallest and comprised only three. Based on the protein sequence analysis of *BnaALMTs* and *AtALMTs*, we analyzed the phylogenetic diversity among them and found that the proteins were subdivided into 5 separate classes (Figure 2). The *BnaALMT* genes were highly variable and had re-arranged with their evolutionary patterns from *AtALMTs*. Based on their genome variation, the two species had lost their portions of their protein sequences during evolution, which suggests that certain of the genes of *BnaALMT* may be evolutionarily similar, but they have missed protein sequences that might have evolved new genes for a specific function or division of the labour (Figure 2). The current reference genome was given as an incomplete assembly and might not cover 100% of the genes of *B. napus.* Darmor-bzh’s reference genome only occupies approximately 79% of the genome of 1130 Mb [28]. We found that some known genes, including *BnaA07g02630D*, *BnaC02g32510D*, *BnaC02g32490D*, *BnaC08g13490D*, *BnaC08g13500D* and *BnaC08g15180D* in Figure 2 were possibly not fully assembled and annotated. If *B. napus* provide a high-quality registry genome, genes with missing sequences or inappropriate assembly can be reviewed and corrected. Moreover, it can be overlooked that modern reference genomes are likely to contain additional *BnaALMT* family members that could miss a partial sequence during evolution, or that an incomplete assembly of the existing reference genomes for *BnaALMT* genes is made available.

### 3.2. Protein Structure of BnaALMTs

The *ALMTs’* protein structures found to date have similar characteristics: five to seven domains in the *n*-terminal region and a long C-terminus, with or without the whole protein up to 50% [15,18,19]. A feature structure study to describe changes in *ALMT* functions with structural changes, including protein truncation, domain transfers and one-point mutations, will provide insight into the functional position of the *n* and C-terminal domains [29]. β-strands paired to form small sheets of ß-sheet share often observed at sequence-specific DNA interfaces, where β-sheets lie flat inside the main groove and lateral chains connect with functional groupings on the edges of the adjacent base pairs on the exposed surface of the sheet [30]. Interestingly, our results of lineage analysis of the *ALMT* family showed only minor improvement on the inclusion of evolutionary information from multiple alignments (Figure S1), indicating that the sequence of evolutionarily related β-strands provides little additional information to distinguish edge and central strand. β-strands paired were formed with *AtALMTs* in sequence alignments of *BnaALMT* genes, and *AtALMT-6,-7,-10,-11,-12,-13* and *AtALMT-14* homologous sequence-specificity consists of β-strands (Figure S1). We analyzed the secondary structures of *BnaALMT* family genes used in our phylogenetic analysis to see which of the secondary structures among the same clade members were conserved or diverged over time. The *BnaALMT* genes possessing diverged structure might have evolved from their homologous to perform varying functions as the secondary structures were highly related to the functions (Figure S1). The higher-order protein structures of the *BnaALMT* genes with *AtATLMTs* (Figure S1) should be investigated for a more detailed study. Conserved sequence WEP was reported by different studies [15,17,18,31], previously. A study demonstrated that three conserved residues in TaALMT1 including E274, D275 and E284 (residue of WEP) are highly conserved throughout the entire *ALMT* family, both Al sensitive and insensitive *ALMTs* [15]. The mutation studies provide evidence that these residues including E284 of WEP are not involved directly in Al response but rather the change in these residues brings conformational changes to the tertiary structure of the protein and hence it causes a change in protein structure. Another study also reported that the residue E284 is part of a WEP fingerprint motif (Trp-Glu-Pro) which is conserved in all *ALMTs* [17] rather than targeting specific residues to investigate the structure-function of *ALMTs* [18]. In our study, we found that WEP is not conserved in all of the proteins of the *ALMT* family. It was missing in some genes and the reason is clear that those genes have either lost a part of the functional gene or those genes have split into two functional genes. Interestingly, the *BnaC08g15180D* gene was lost in motif function analysis. WEP had also mutated into WCR in only one gene of the *BnaALMTs* family. Looking into the functional characterization of those mutated genes in *B. napus* will also shed a light on the function of WEP. The function of WEP as a whole will probably be different from the function of E284 alone but the claim needs to be proved through experimental studies. The whole sequence of WEP needs to be mutated to get a clear picture of WEP rather than the function of E284.

### 3.3. Expansion of the ALMT Family in Brassica napus

Genome duplication was reported as a major factor in the growth of eukaryotic plants and animals [32]. The primary causes of gene duplication are often known to be whole-genome duplication [33]. In the *B. napus ALMT* gene evolutionary relationship classification two *Arabidopsis thaliana* members, *ALMT-11* and *ALMT-12* were found evolutionary parallel to each other in a tree (Figure 1) and might have arisen by gene duplication. Gene duplication is known to be one of the key drivers of genomic and genetic evolution. Thirty-nine *BnaALMT* genes (87.8%) were predicted to have syntenic connections in the *B. napus* genome with *BoALMTs* (30 genes) and *BrALMTs* (18 genes) in the current study. *BnaALMT* genes in A and C sub-genomes of *B. napus* are closely related to *ALMT* genes in the A sub-genome of *B. rapa* and C sub-genome of *B. oleracea*, respectively (Figure 4 and Figure 5). These findings indicated that the expansion of the *ALMT* family in *B. napus* was mainly caused by allotetraploids (Figure 5). Additionally, there were 129 segmental duplication occurrences of 25 of the 39 *BnaALMT* genes, while only nine tandem duplication occurrences were found (Figure 4). *Brassica* species, unlike the *A. thaliana*, experienced an extra-genomic tripliment, which contributed to the gene production and the diversification of *Brassica* plants [28]. In principle, therefore, three orthologs in *B. rapa* and *B. oleracea* should equate to one *Arabidopsis* gene, while *B. napus* should produce six syntenic copies in each *Arabidopsis* gene as it has been derived from the recent hybridization between *B. rapa* and *B. oleracea* [28]. In the present research, expansion of the *BnaALMT* genes in *Arabidopsis* resulted in the genes in *B. napus* more than two times and in *B. rapa* and *B. oleracea* one to two times more genes (Figure 5). A surprisingly significant number of family members have suggested that substantial replications, which also happened during development, have largely enlarged and rearranged the genome [34]. Polyploidization of genomes usually is followed by mass rearrangements of chromosomes [35]. *ALMT* genes were distributed in *B. napus* across 15 chromosomes, although the dispersion varied from *B. napus* to *B. rapa* sub-genome A to *B. napus*, to the C sub-genome of *B. napus* to the *B. oleracea* sub-genome (Figure 5) that indicates that during allopolyploidization and domestication large diversification and chromosomal rearrangements occurred.

### 3.4. Expression Profile of BnaALMT Genes in Response to P Deficiency

The low P availability in many soils is a widespread adversity that often restricts plant growth and development because phosphates are strongly fixed in sparingly soluble complexes [36]. A possible mechanism for enhancing the use of sparsely soluble P forms is the secretion of malate in plants by the Al-activated malate transporter (*ALMT*) gene family. In this study, thirty-nine members of the *BnaALMT* genes family have various expressions in the different tissues (seed, silique, flower, root, stem and leaf) of the *B. napus* cultivar “Zhongshuang 11” (Figure 6). Of them, 18 *BnaALMTs* were mainly expressed in roots; while 11 were more leaf expressed than any of other tissues (Figure 6).

Nearly half of the *BnaALMT* members were up-regulated in the roots with a few (*BnaCnng04670D*, *BnaA07g02630D*, *BnaC07g34970D*) in leaves by P-deprivation, although four (*BnaC08g13480D*, *BnaA08g19430D*, *BnaA09g28580D*, *BnaC03g31660D*) genes had shown similar expression levels in both P sufficient and deficient supplies. These observations indicated that different members of the *ALMT* family might perform different roles in plants in responding to P deficiency. The application of P in shallow lateral roots in a divided root system under Al-stress in hydroponics was observed to facilitate a malate exudation of genotypes of P-efficient soybean (glycine max), which was also related to superior Al-tolerance [37]. Twenty-six of 34 *GmALMTs* show up-regulated in root, leaf and flowers of soybean under phosphate starvation [1]. Pi starvation also greatly improved *CsALMT1′s* expression in *Citrus sinensis* [38]. Further analysis is required in *B. napus* cultivar “Zhongshuang 11” to investigate the role of the *ALMT* genes by producing their overexpression materials or Crispr-CAS9 mutants.

## 4. Materials and Methods

### 4.1. Materials

*B. napus* cultivars “Zhongsuang11” were used for this study. The seeds are provided by the Oil Crops Research Institute, Chinese Academy of Agriculture Science, Wuhan, China.

### 4.2. Identification and Extraction of ALMT Genes

There were 14 *ALMTs* in Arabidopsis and they were designated as from *ALMT1* to *ALMT14*. The full-length genomic sequences, CDSs and protein sequences of *BnaALMTs* were retrieved from the *B. napus* genome database (http://www.genoscope.cns.fr/brassicanapus/; accessed on 11 April 2020) by BLAST using *AtALMTs.* The homologous genes of the entire family of *ALMT* genes were used to confirm that all the genes belonged to the family of *ALMT* through *B. napus* cultivar “Zhongshuang11” [23].

### 4.3. Multiple Alignments, Phylogenetic and Bioinformatics Analysis

Multiple sequence alignments of the *ALMT* protein sequences in *Arabidopsis* and *B. napus* were performed using ClustalW. All the *BnaALMT* protein sequences along with *AtALMTs* were subjected to Geneious software for phylogenetic tree, evolutionary relationship and gene structure analysis, and all those sequences that clad with *AtALMTs* were considered as *BnaALMTs*. The Phylogenetic tree and its secondary structures were constructed using MAFFT alignment and FastTree plugins in Geneious 11. For the construction of the tree, *ALMT* gene variations and secondary structures were used to retrieve the related sequences from NCBI. Duplicates and poorly aligned sequences were removed before constructing these figures. Gene duplication data were retrieved from Plaza 4.0 (https://bioinformatics.psb.ugent.be/plaza/; accessed on 15 April 2020), and conserved motifs were identified through MEME online suit (http://meme-suite.org/tools/meme/; accessed on 15 April 2020) with the default setting.

### 4.4. Chromosomal Location and Gene Duplication Analysis

Physical location information of the *BnaALMT* genes was retrieved from the CNS-Genoscope genomic database (http://www.genoscope.cns.fr/brassicanapus/; accessed on 16 April 2020) and was mapped to *B. napus* chromosomes using Circos [39]. Gene duplication events and collinearity relationships were analyzed using the Multiple Collinearity Scan toolkit (MCScanX, http://chibba.pgml.uga.edu/mcscan2/; accessed on 15 April 2020) [40]. The chromosome location information of the *ALMT* genes in *Brassica* species (*B. napus*, *Brassica rapa* and *Brassica oleracea*) were retrieved from the (http://www.genoscope.cns.fr/brassicanapus/; accessed on 18 April 2020) database and Ensemble (http://plants.ensembl.org; accessed on 18 April 2020). The MapInspect software was used to draw the gene chromosome location diagrams.

### 4.5. Transcriptional Profile of BnaALMT Family Genes

Tissue-specific expression data of the selected 39 *BnaALMT* genes were downloaded from National Genomics Data Centre (CRA001775) available online (http:bigd.big.ac.cn/; accessed on 19 May 2020). Gene expression levels were determined according to a previous study by [23]. The data were subjected to the “expression-based heat maps” tools of heat mapper, an online tool for converting numerical data into heat maps (http://www2.heatmapper.ca/; accessed on 23 May 2020). Heat maps were built using the row as scale type, clustering method as average linkage and distance measurement method as Euclidean.

The seedlings of *B. napus* cultivar “Zhongshuang11” were planted in Hoagland solution in an illuminated growth room (300–320 μmol m^−2^ s^−2^; 24 ℃ daytime/22 ℃ night; 16 h light/8 h dark) in three repeats for 14 days during June, 2020. The solution contained Ca (NO_3_)_2_ 5.0 mM, KNO_3_ 5.0 mM, KH_2_PO_4_ 0.25 mM, MgSO_4_·7H_2_O 2.0 mM, H_3_BO_3_ 46 µM, MnCl_2_ 9.0 µM, CuCl_2_ 0.3 µM, ZnCl_2_ 0.8 µM, NaMoO_4_ 0.32 µM and EDTA-Fe 50 µM. For P deficiency treatment, KH_2_PO_4_ was replaced by K_2_SO_4_. The q-PCR was performed for selected twenty-two genes to further validate RNA-seq data using LioBIO-RAD CFX96 qPCR detection system. The RNA-seq sequencing libraries were subsequently sequenced using Illumina HiSeq 2000 platform (Illumina, Salt Lake City, UT, USA) and the transcript abundance (FPKM value) of each gene was calculated based on the length of the gene and the reads mapped to that gene [41]. The FPKMs values of genes were used to produce a heat map using the program (http://www2.heatmapper.ca/; accessed on 28 May 2020). The roots (R) and leaves (L) of *B. napus* cultivar “Zhongshuang11” were individually harvested, and each sample included three independent biological replicates. The frozen samples were taken from −80 °C and were crushed into a fine powder. The total RNA of each sample was extracted using an RNA extraction kit (BioTeke, Beijing, China) according to the manufacturer’s recommendations. RNA set was extracted using Trizol reagent (Tiangen Biotech (Beijing) Co., Ltd., Beijing, China) and then treated with gDNA digester (Tianjin Novogene Bioinformatics Technology Co., Ltd. www.novogene.com; accessed on 19 June 2020) to remove the leftover genomic DNA. First-strand cDNA was synthesized from the total RNA using HonorTM II 1st Strand cDNA Synthesis SuperMix for qPCR (gDNA digester plus) kit provided by Novogene. q-PCR was performed using Unique AptamerTM qPCR SYBR^®^ Green Master Mix (No Rox) provided by Novogene (Tianjin Novogene Bioinformatics Technology Co., Ltd. www.novogene.com; accessed on 19 June 2020). The melt–curve analyses were performed using the conditions previously used. The obtained ~6 Gb Illumina cleaned reads were aligned with reference Darmor-bzh genome [28]. Actin was used as the internal reference gene [42]. Specific primers were used for determining the expression level of the selected *BnaALMTs* in time series samples of treated plants (Table S1).

### 4.6. Statistical Analysis of Data

Statistics were performed by using Student’s *t*-test. Significance of differences was defined as * *p* < 0.05 and ** *p* < 0.01.

## 5. Conclusions

In summary, a total of thirty-nine *BnaALMT* genes were identified and were clustered into five branches in the phylogenetic tree based on protein sequences. The *BnaALMT* gene family shared high similarity with their Arabidopsis orthologues, which suggested that the *BnaALMT* genes family were evolutionary, conserved and each gene within the subfamily may share high similarity in biochemical and biological functions with other genes of the same subfamily in Arabidopsis. The distribution of *BnaALMT* genes in A and C sub-genomes of *Brassica* species and their syntenic relationships suggested that whole-genome duplication (polyploidy) and segmentary duplication were the main factors to extend the *ALMT* family of *B. napus*. Nearly half of the *BnaALMTs* were up-regulated after P deprivation in the roots. Further studies on mechanisms underlying the genotypic differences in *BnaALMTs* expression will facilitate the breeding of *B. napus* varieties with improving P use efficiency.

## Supplementary Materials

The following are available online at https://www.mdpi.com/article/10.3390/ijms22094625/s1, Figure S1: The evolutionary history of beta-strand. Table S1: Specific primers were used for determining the expression level of the selected *BnaALMTs* in time series samples of treated plants. Figure S2: Expression patterns of the up-regulated *BnaALMTs* at a P sufficient supply and a P deficiency supply by Hoagland’s solution. Table S2: Primer sequences used in the qPCR experiment.
