# Supplementary material for: Genome-Wide Analysis, Evolutionary History and Response of ALMT Family to Phosphate Starvation in Brassica napus
Source: Int J Mol Sci. 2021 Apr 28;22(9):4625. doi: 10.3390/ijms22094625 (PMC8125224; doi:10.3390/ijms22094625)
Supplement: Supplementary file 1 [file ijms-22-04625-s001.zip › ijms-1194775-supplementary.pdf]

Supplementary Files

Table S1

Primer Name	Sequences (5′-3′) Forward Primers	Reverse Primers
Actin	ACAGTGTCTGGATCGGTGGTTC	TGCCTCATCATACTCAGCCTTG
BnaC08g13520D	AGCAACGTTCGTGAGGTTCT	AGCAACGTTCGTGAGGTTCT
BnaC08g15170D	ACTTGGACACAGTCTCCCAC	TGTCCCCATACTCTCTGGCT
BnaC05g06110D	TGCAATGTGGGCTGTCATGA	GTTGGACCGGACAAACTTGC
BnaC08g13490D	TGCAATGTGGGCTGTCATGA	GTTGGACCGGACAAACTTGC
BnaC08g13540D	AATCGACCGTGTCATCCGAG	AGTCCACGGAACTCTCTCCA
BnaA06g04860D	TGCAATGTGGGCTGTCATGA	GTTGGACCGGACAAACTTGC
BnaC05g14120D	GTACGTGAGAGTAGGTGGCG	ACTTTAGCTCCTTCCACGCC
BnaC08g37290D	GGTCGAGGATGAGGAGGAGT	GCTTCGAAATGGGCGACTAC
BnaA06g12560D	GATCCAGAGCCAGCAATCGA	GCTAGAGACAGAGCACTCGC
BnaCnng04670D	TCCAGCCTCTTCCTCGATGA	GGCGAAGTAAACTTTCCGGC
BnaA07g02630D	GGTCCAGAGCAAAGCAGAGA	AGCCATTCCCATCTTGACGG
BnaA05g27400D	AGCGAATCTTGCGAGATGGG	GGTGCCTTAGCGTCAGAGAG
BnaC03g74300D	AGCTTTGGGGTTTCCGGTAT	TCGAGCAAGGTGTACTGCAC
BnaC05g41520D	GGCGAATCTTGCAAGATGGG	GGTGCCTTAGCGTCAGAGAG
BnaA01g26740D	CGAAACAGGTATCGTGGCCT	TCAACTCCTCGAACGCGTAG
BnaA02g20300D	AGCCTAGTCTCAGTCGGAGG	AAGGCGGTTAAGCTGAGATCC
BnaA03g26740D	GTTGCAACGAACAAACCCGA	TTCATCCACTGCTTCGACCG
BnaC02g27820D	TGGGACAACCGAGAGATTGG	GCTTTTGCTGGATCATCTGCC
BnaC02g32490D	CGTCGACGGCAATGTTTTGA	CTCTTCTCTCCAACCGGTCC
BnaC02g32510D	GTTCTCGAGTTCTCTGCCGG	TACAGCAGCGCCAATGAAGA
BnaA06g40390D	TTACCGTTTGCTGCCTTTGC	GACTCGAACCTCCACATCCG
BnaC07g19290D	CGTTACCGTTTGCTGCCTTT	CGACTACCTCCACATCCGTC

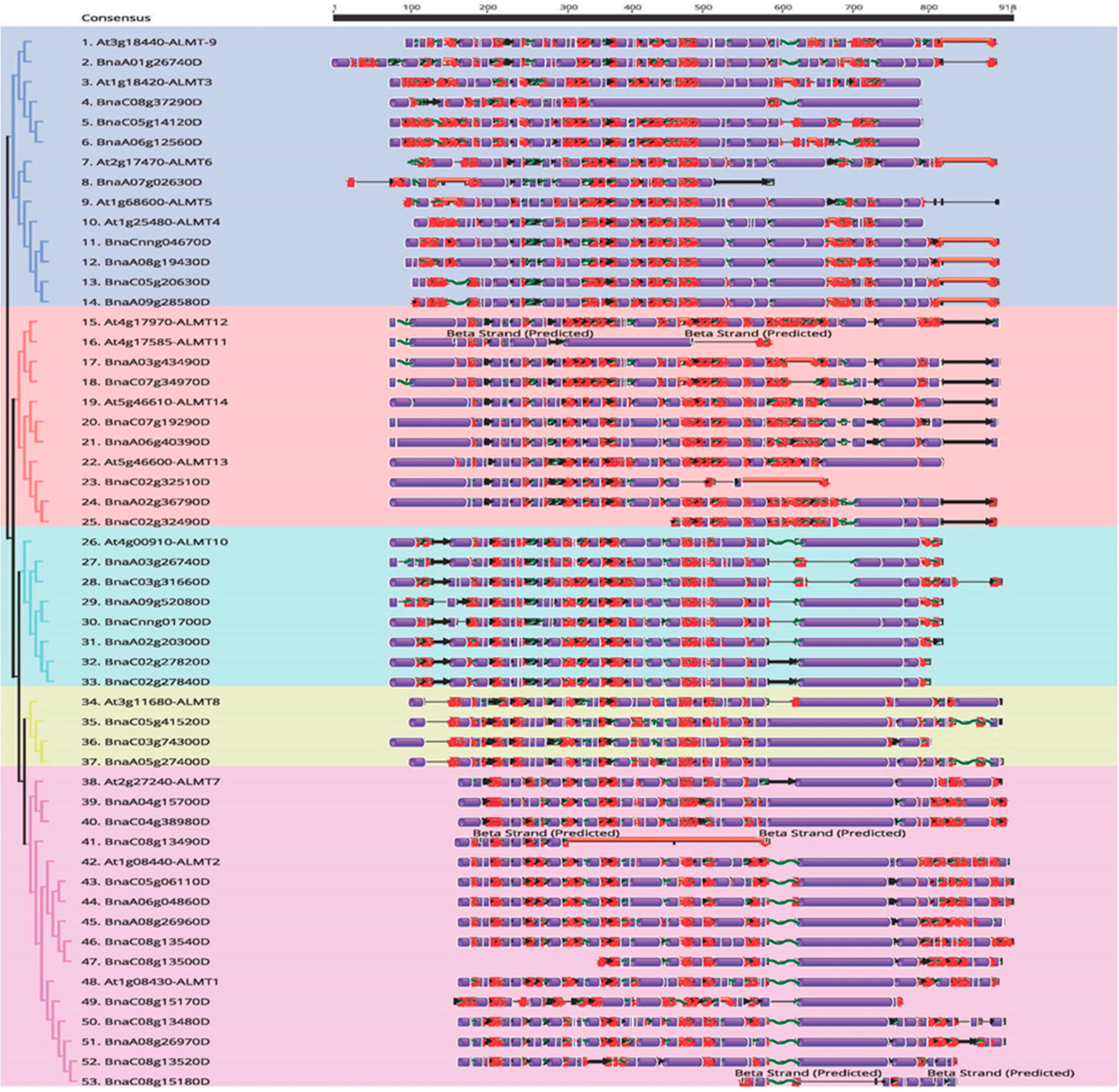

Figure S1. The evolutionary history of beta-strand. Gene structures of ALMT genes family according to their phylogenetic relation between Brassica napus and Arabidopsis thaliana tree was constructed by Genius 11. Given perfect knowledge of the positions of β-strands in a protein sequence, edge strands can be distinguished with reasonable accuracy from central strands. Here we consider the effect of using predicted secondary structures. Starting from ancestral pools of β-strands, each group contains beta-strands. The protein secondary structure is divided into three states of coil, alpha-helix and turns. The black arrows show Beta strands, purple-blue represents Alpha helix, Coil is green single DNA strand and red indicates turns, which serve to reverse the direction of the polypeptide chain. The length of the scale represents base pairs (bp) length which is placed at the top.

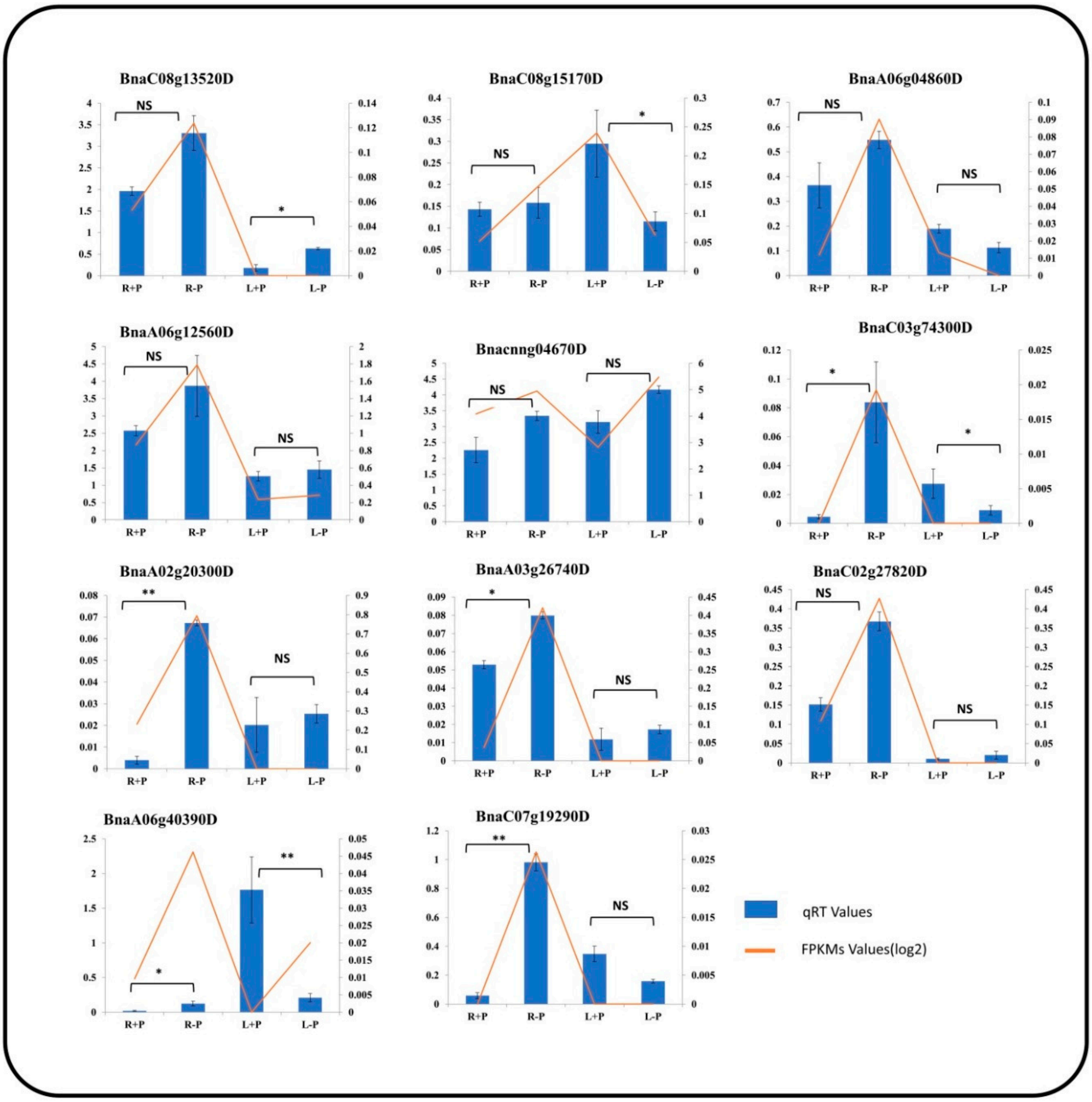

Figure S2. Expression patterns of the up-regulated BnaALMTs at a P sufficient supply and a P deficiency supply in Hoagland's solution. The same plants and samples were used for q-PCR measurements with three biological replicates. The relative expression of BnaALMTs in the Brassica napus root and leaf under contrasting P supplies was quantified by q-PCR; The bars represent q-PCR data while the line represents RNA-seq data. Student T-test was applied to treated and non-treated tissues (leaves and roots) to find out the statistical differences caused by phosphorous application in the tissues. ns= non-significant, *= significant at 0.05, ** = significant at 0.01 and *** = significant at 0.001. All primer sequences are listed in supplementary table S1.
